# Molecular bridge-mediated ultralow-power gas sensing

**DOI:** 10.1038/s41378-021-00252-3

**Published:** 2021-03-29

**Authors:** Aishwaryadev Banerjee, Shakir-Ul Haque Khan, Samuel Broadbent, Ashrafuzzaman Bulbul, Kyeong Heon Kim, Seungbeom Noh, R. Looper, C. H. Mastrangelo, H. Kim

**Affiliations:** 1 Department of Electrical and Computer Engineering, University of Utah, Salt Lake City, UT 84112 USA; 2 Department of Chemistry, University of Utah, Salt Lake City, UT 84112 USA; 3 Gyeongsang National University, Jinju, South Korea

**Keywords:** Sensors, Nanosensors

## Abstract

We report the electrical detection of captured gases through measurement of the quantum tunneling characteristics of gas-mediated molecular junctions formed across nanogaps. The gas-sensing nanogap device consists of a pair of vertically stacked gold electrodes separated by an insulating 6 nm spacer (~1.5 nm of sputtered α-Si and ~4.5 nm ALD SiO_2_), which is notched ~10 nm into the stack between the gold electrodes. The exposed gold surface is functionalized with a self-assembled monolayer (SAM) of conjugated thiol linker molecules. When the device is exposed to a target gas (1,5-diaminopentane), the SAM layer electrostatically captures the target gas molecules, forming a molecular bridge across the nanogap. The gas capture lowers the barrier potential for electron tunneling across the notched edge region, from ~5 eV to ~0.9 eV and establishes additional conducting paths for charge transport between the gold electrodes, leading to a substantial decrease in junction resistance. We demonstrated an output resistance change of >10^8^ times upon exposure to 80 ppm diamine target gas as well as ultralow standby power consumption of <15 pW, confirming electron tunneling through molecular bridges for ultralow-power gas sensing.

## Introduction

The development of low-power Internet-of-Things (IoT) sensor systems has been rigorously pursued by an increasing number of scientific communities to enable continuous access to various information around the globe. Detection of motion, humidity, and temperature was proposed for the purpose of creating a user-accessible database via the implementation of a low-power IoT wireless sensor network by Laubhan^1^. Improvements to gas detection techniques have been rigorously pursued to cover a wide range of commercial IoT applications, including indoor air quality monitoring, and industrial applications, such as the detection of hazardous or combustible gases. Additionally, similar gas sensors have been deployed for remote and continuous environmental monitoring to detect the presence of toxic gases such as volatile organic compounds (VOCs), CO, SO_2_, H_2_S, and O_3_^2^.

Among such power-limited IoT applications, gas sensors remain one of the most challenging components due to the power-hungry nature of gas sensing, especially in the required continuous operation mode. Although gas sensors typically vary in their operating temperature requirements, most of them consume >10 μW of power due to the requirements of elevated temperature or heating^3^. Recently, some low-power (sub-10 μW) gas sensors have been demonstrated; however, they displayed only limited output signal changes of less than a few orders of magnitude, ultimately being limited in the minimum detection amount when utilizing ~μWatt level of power. These sensors achieved low-power operation by utilizing self-heating under a bias voltage or by minimizing electrical leakage paths within the device, such as self-heating carbon nanotubes^4^, Si-nanowire/TiO_2_ core–shell heterojunctions^5^, chemically gated Si nanowire/SnO_2_ thin film FETs^6^ and Pd-based Si nanomembrane sensors^7^. They were limited in the output signal range mainly because the transducer output varied linearly with the analyte concentration in a physically connected device structure. For example, a CNT NO_2_ sensor displayed only a < 10 times change in the output current upon exposure to 0.9 ppm target gas while consuming 0.8 μW of DC power, and an FET gas sensor produced an output of <10^2^ times upon exposure to 104 ppm analyte while consuming ~1 μW of DC power.

Such limited output signals can be further amplified without additional power consumption by physically forming an electrically conductive bridge across an otherwise electrically nonconducting open space mediated by the capture of target gas molecules. This capture would lead to electron tunneling through the molecular bridge, resulting in a current change of multiple orders of magnitude due to the exponential dependence of the tunneling current on parameters such as the tunneling distance and energy barrier. An open-air gap could be realized by constructing a parallel plate structure between metal electrodes and matching the gap distance with the molecular size of the target gas. The initial air gap provides an extremely high DC off resistance between electrodes. This leads to a minimal leakage current and hence a highly reduced standby power consumption. A similar nanogap concept was previously used but limited to in-liquid analysis and detection of biomolecules such as proteins and DNA^8–14^. We previously introduced the concept for quantum tunneling-based gas sensing with preliminary results^15–25^.

This paper reports detailed proof-of-concept results of a new family of ultralow-power nano-air-gap sensors based on molecular bridge formation across nanogap electrodes. These air-gap sensors evidently demonstrated very high ON/OFF output signal ratios due to molecular bridge-mediated electron quantum tunneling. Specifically, this article reports the operation principle, device structure and fabrication, and sensor test results.

## Materials and methods

### Device design and fabrication

**Fig. 1:**
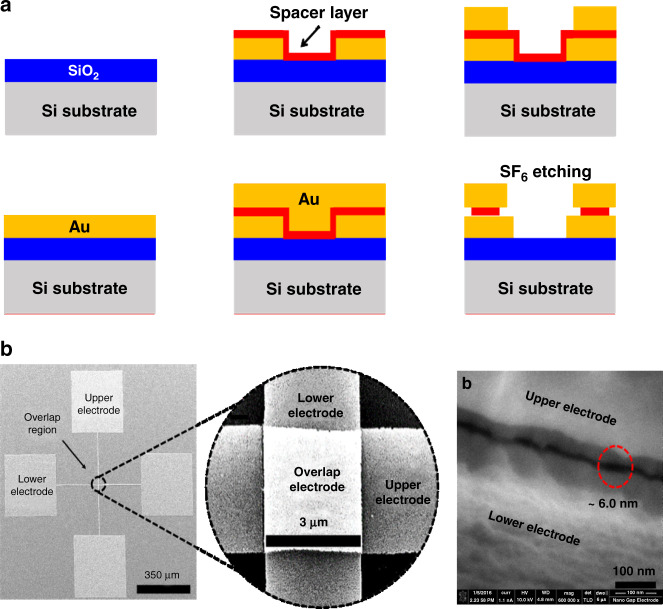
Fabrication flow and high resolution SEM images of nanogap devices. **a** Simplified fabrication flow of nanogap electrodes. The fabrication process began by (1) growing 500 nm of thermal oxide on a Si wafer. (2) Then, 200 nm of Au (with a Cr adhesive layer) was deposited and patterned to define the lower electrodes. (3) Next, a SiO_2_ layer (~4.5 nm) was deposited using plasma-enhanced ALD at a 200 °C chuck temperature, and an additional α-Si layer (~1.5 nm) was deposited, which together determined the thickness of the spacer layer. The thickness of atomic layer deposition (ALD) SiO_2_ and DC sputtered α-Si was verified using ellipsometry (Woollam Variable Angle Spectroscopic Ellipsometer (VASE)), and the thickness of the entire spacer layer was verified using SEM imaging. (4) On top of the spacer layer, an upper Au electrode layer (200 nm) was sputtered and then (5) subsequently patterned using standard lithographic techniques. Finally, (6) the spacer stack layers were etched away through SF6 plasma dry etching, thereby forming an air gap along the edges of the top electrode. **b** SEM images of the fabricated device, where the overlap area was reduced to suppress parasitic current flow. The device footprint was 0.36 mm^2^, and the overlap area was ~16 μm^2^ (middle). The nanometer-scale dimension of the air gap formed between the upper and lower electrodes was confirmed by high-resolution SEM imaging

Details of the device fabrication method have been extensively discussed in our previous article^26^. Figure a shows the simplified fabrication process flow. The nanogap dimension is defined by the thickness of the deposited spacer layer and is suitably engineered to match the length of the linker molecule and the target molecule for successful and specific capture of the target analyte. Figure 1b shows SEM images of the fabricated nanogap device and overlap area and a high-resolution SEM image of the nanometer-scale air gap.

### Structure of target and linker molecule

**Fig. 2:**
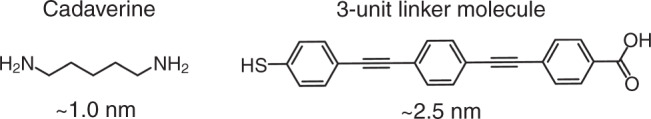
Chemical structures and molecule lengths of the target molecule (on the left) and linker molecule (on the right). The IUPAC name of the linker molecule is (4-((4-((4-mercaptophenyl)ethynyl)phenyl)ethynyl)benzoic acid)

To demonstrate the validity of the proposed sensing mechanism, we aimed to detect a vapor of 1,5-pentanediamine, commonly known as cadaverine, as the target gas molecule. Cadaverine was purchased from Millipore Sigma in liquid form (97% purity). Figure S1 in Supplementary section describes the synthesis of the linker molecules. Figure 2 shows the chemical structures of the target gas, cadaverine, and the synthesized linker molecule. The thiol linker molecule is comprised of three components: the sulfur head group, the aromatic and alkyl spacer chain, and the benzoic acid tail group. The head group, consisting of thiol molecules (-SH), is capable of covalently bonding to the gold surface. The spacer chain defines the length of the linker molecule and, due to its conjugated nature, allows augmented electron flow throughout the molecule. Capture of our targets is achieved by the tail segment of the linker, which in this case is composed of benzoic acid. The terminal carboxylic acid from the benzoic acid and the matching length of the spacer chain with the nanogap distance together determine the specificity of the target gas molecules to be captured. The IUPAC name of the synthesized linker molecule is (4-((4-((4-mercaptophenyl)ethynyl)phenyl)ethynyl)benzoic acid). Additional details of the SAM linker molecule structure and synthesis are provided in the supplementary section.

### SAM coating procedure, coating characterization, and target capture specifics, and linker conductivity measurements

**Fig. 3:**
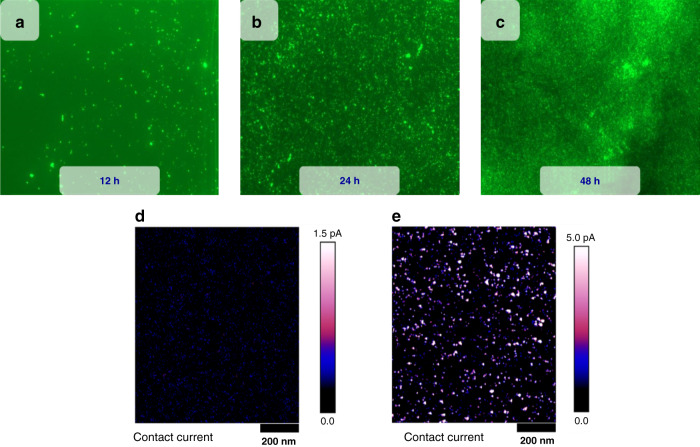
SAM coating procedure, visual confirmation of optimum immersion parameters, and qualitative comparison of the conductivity of the synthesized linker molecule and commercially available alkane-thiol linker molecule. **a**–**c** Cr/Au-coated samples functionalized for 12, 24, and 48 h and exposed to similar concentrations of fluorescent cadaverine for 2 min. The lighter spots in the image are representative of successful capture of fluorescent cadaverine molecules by the SAM coating. As shown in the figure, the sample immersed for 48 h displayed the highest density of cadaverine-to-linker capture, resulting in a 48-h immersion period as our standard functionalization protocol. **d**, **e** To compare the conductivity of our synthesized linker molecules (with a thiol end group) with that of commercially available alkane thiols (which are inherently nonconductive), we fabricated separate Cr/Au-coated glass samples functionalized with (1) our synthesized linker molecule and (2) 16-mercaptohexadecanoic acid, a commercially available alkanethiol purchased from Millipore Sigma. As shown in the figure, samples functionalized with our synthesized linkers qualitatively exhibited augmented conductivity compared to the samples functionalized with the commercially available alkane-thiol linker molecule. The conductivity was measured using Peak force tunneling atomic force microscopy (PF – TUNA, Bruker). The lighter color spots are indicative of a higher current reading by PF-TUNA and are hence an indication of higher conductivity

Thiol SAM chemisorption on gold surfaces, first reported by Bell Laboratories in 1983^27^, was performed by dissolving 30 mg of the linker chemical in 5 mL of dimethyl sulfoxide (99.9% purity as purchased from Millipore Sigma) and ~15 mL of ethanol and immersing the gold-coated device for a specified amount of time. It should be noted that ethanol does not chemically influence the coating procedure, and the required volume is simply to ensure complete immersion of the device within the solution. Figure 3a–c shows images taken under a fluorescence microscope of a sample exposed to similar concentrations of the fluorescent cadaverine target (Alexa Fluor 488, Sigma Aldrich) for 2 min after being immersed in the linker chemical solution for different periods of time (12, 24 and 48 h). Figure 3c shows the highest density of captured fluorescent cadaverine molecules and, by extension, the formation of an SAM upon immersing the sample for 48 h. Leaving the sample submerged within the solution for more than 48 h resulted in the gold layer peeling off and was therefore avoided. Hence, a 48-h coating procedure was chosen to be part of the protocol. Conductivity measurements of the linker solution performed using Peak force tunneling atomic force microscopy (PF-TUNA) revealed higher conductivity than commercially available alkane-thiol linker molecules, as shown in Fig. 3d, e.

### Estimation of the target gas concentration

The concentration of the cadaverine target gas was estimated by first establishing a calibration curve between known cadaverine liquid volumes and corresponding signal intensities (areas) and then comparing the obtained calibration curve to the signal intensities obtained by injecting an unknown concentration of the gas sample. Here, a gas chromatography-flame ionization detector (GC-FID) system was utilized to inject different masses of liquid cadaverine and monitor the chromatogram signal intensities at each mass. The utilized masses of cadaverine were 6.2, 12.4, 18.6, 24.8, and 31 ng, with corresponding diluted cadaverine samples of 0.1, 0.2, 0.3, 0.4, and 0.5 μL. Note that the cadaverine sample was diluted to 1% with a mixture of water to reach a much lower level (nanogram) of liquid injection and, more importantly, to maintain the signal intensity in a similar range as later gas injection. Finally, the injected mass (*m_inj*) vs. signal intensity (*A_FID*) data were fitted with a linear equation: *A_FID* = 31.18×*m_inj*-0.03794.

To determine an unknown concentration of cadaverine, first, a gas sample was prepared inside a closed chamber, and then, by injecting the gas sample into the same GC-FID system, the mass of the gas sample was calculated according to the above equation. The calculated mass was then converted to ppm considering complete evaporation of cadaverine at STP. For example, if the estimated mass was 1.3 ng, this corresponded to 12.31 picomoles of liquid mass or 301 pL of gas at STP. For this example, the gas injection volume was 100 µL, and therefore, the ppm was calculated as 3.01 ppm.

### Sensor measurements in the test chamber

**Fig. 4:**
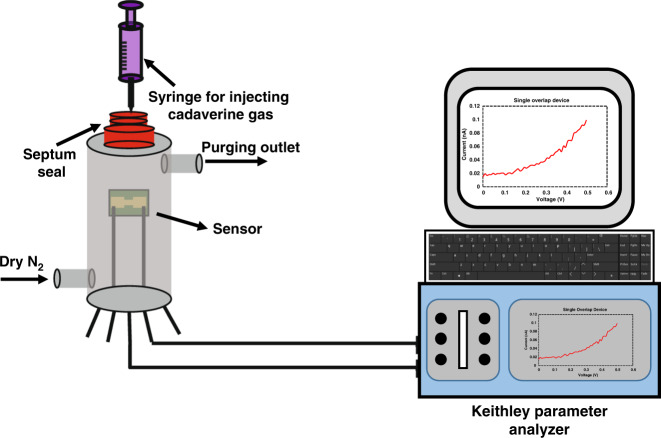
Experimental setup for sensor measurements. The nanogap sensor is placed within a steel gas testing chamber that has a septum seal and purging outlets. Cadaverine is injected into the chamber through the septum seal, using a syringe. The chamber has an electrical feedthrough, connected to the sensor, to measure the dynamic resistance of the sensor after exposure to analyte, using a Keithly 4200A-SCS parameter analyzer.

The test setup utilized a stainless steel cylindrical structure (height: 15 cm and diameter: 5.5 cm) with an injection hole sealed with a rubber septum seal and two electrical feedthroughs. The two electrical feedthroughs connected to the fabricated sensor located inside the chamber were also connected to an impedance analyzer, a Keithley 4200A-SCS, outside the chamber. The Keithley impedance analyzer had a resolution of 100 fA. The chip was supplied with a DC bias of 0.7 V across the nanogap-separated electrodes. The ambient test conditions were continuously monitored using a BME280 chip, which is a commercially available humidity, temperature, and pressure sensor^28^. The chamber was not subject to any vacuum modifications, and it was kept under typical atmospheric conditions at atmospheric pressure and maintained at a room temperature of ~23 °C during all tests. A µ-syringe was used to introduce cadaverine gas into the test chamber through the septum seal. The concentration of cadaverine within the chamber was calculated from the volume of cadaverine injected as described in Section 2.4, and the sensor response was measured using the Keithley impedance analyzer. Figure 4 shows a schematic of the test setup.

### Tunneling operation based on molecular bridging

**Fig. 5:**
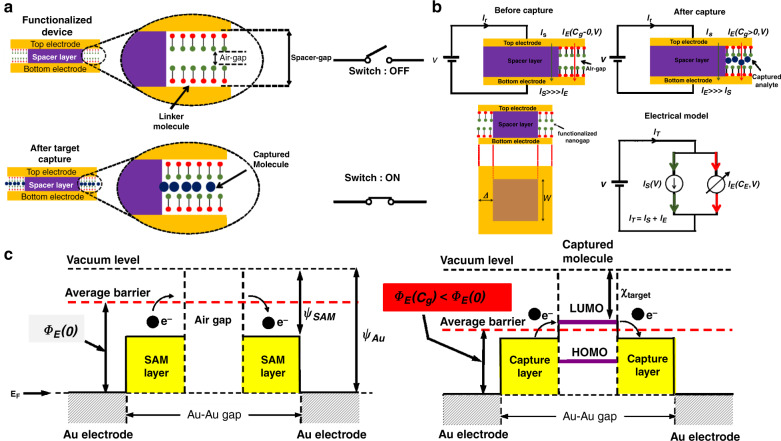
Schematic representation of the working principle of the nanogap sensor, equivalent electrical model, and representative band diagram. **a** Schematic of the nanogap device after fabrication and analyte capture. Successful target capture turns the switch ‘ON’. **b** Schematic representation of current conduction before and after analyte gas capture, and equivalent electrical model depicting the two current conducting paths. **c** Average edge energy barrier in the absence of a target analyte gas, and **d** average energy barrier at a molecular junction established by the capture of a target gas molecule. The average energy barrier is lowered, leading to a larger tunneling current. *I*_*S*_ is the electric current through the dielectric spacer stack, and *I*_*E*_ (*C*_*g*_) is the net current through the molecular bridges formed due to target gas capture and is a function of the target gas concentration *C*_*g*_. Φ_*E*_(*0*) and Φ_*E*_(*C*_*g*_) are the average potentials barrier before and after the capture of the target gas, respectively. χ_target_ is the electron affinity of the captured target. ψ_SAM_ and ψ_Au_ are the work functions of the SAM layer and the metal layer, respectively

Figure 5a illustrates the sensor operating principle based on molecular bridging. The two electrodes are originally disconnected, resulting in a high tunneling resistance on the order of 10^9^ Ω as the ‘OFF’ state. These electrodes are coated with a self-assembled monolayer (SAM) of conjugated thiols that capture the target gas molecules. After molecular capture, additional current conduction paths are created between the electrodes via the formation of capture-mediated molecular bridges. This leads to an enhanced quantum tunneling current and a significant reduction in the junction resistance as the ‘ON’ state.

The electrical *I*–*V* characteristics and device resistance are determined by the species trapped in the nanogap, which can be two organic SAM layers separated by an air gap (OFF state) or an SAM-captured gas molecule-SAM chain (ON state). The electrical conductivity of these is examined below.

#### Thiol SAM capture layer

Generally, the electrical properties of organic molecules can be determined by using AFM/STM^29,30^, or (mechanically controllable breaking) MCB techniques^31–34^ or can be simulated using computational chemistry methods^25–27^. Electrical transport through conventional alkane(di)thiol SAMs has been described as pure quantum tunneling across a thin dielectric film that has a rectangular potential barrier with image charge effects included, as described by J. Simmons in 1963^35^. The dielectric constant of a typical alkane-thiol SAM layer was determined by impedance measurements to be 2.1 according to Akkerman^36^. Akkerman also verified that the barrier height of alkane-thiol SAMs sandwiched between a pair of Au electrodes and protected by a layer of PEDOT:PSS (within the spacer stack) was in the range of 4–5 eV. Although alkane thiols are poor conductors of electricity, the conductivity of thiol molecules can also be engineered by the addition of alternative alkynes and aromatic rings within the molecule itself. This modification leads to a conjugated molecular arrangement that results in amplified charge transport along the entire molecule because of delocalized π-electron orbitals, which can freely move along the whole molecule. Bower also verified that conjugation in SAMs containing oligophenyl groups led to a reduction in the attenuation constant *γ*, which essentially meant an augmented conductance for molecules having a higher degree of conjugation^37^. This exponential dependence of the resistance of SAMs on the SAM length ($R_{SAM} = k\cdot e^{(\gamma \cdot length)}$) has been extensively studied and verified. Using a combination of the non-equilibrium Green’s function, Density functional theory (DFT), and confirmed *I*–*V* characteristics, Ratner^38^ concluded that the degree of conjugation and overlap of the orbital densities of the molecular end group and the contact electrodes together determine the heightened conductivity of these molecular chains because of constant delocalized charge transport. Alkane diamine molecules have also been studied using common computational chemistry algorithms^39^, where the length dependence of the electrical conductance has been observed to be exponential, similar to thiol linker molecules. Generally, most organic molecules demonstrate an exponential dependence of the electrical properties on the molecule length. However, it must be noted that there is a transition molecular length beyond which the resistance dependence is almost ohmic in nature. Although this critical length depends on the specific organic molecule forming the molecular chain, generally, for conjugated molecules, it is ~4.0 nm^40^. This exponential dependence of the electrical resistance on the length of the molecular chain is a lower limit of the device resistance; hence, for higher resistance changes, one should engineer the nanogap and SAM dimensions such that the SAM is as short as possible and the air gap is the same length as the captured gas molecule. This combination maximizes the resistance ratio *R*_*OFF*_*/R*_*ON*_ and the dynamic range of the device. The electrical equivalent model for the device is discussed below.

#### Electrical equivalent model

The mechanism for electrical current conduction across the junction of our device is considered to be electron tunneling. In this device, as shown in the schematic of Fig. 5b, there are two possible electron conduction paths represented by current sources *I*_*S*_ and *I*_*E*_*(C*_*g*_*)*. Current *I*_*S*_ represents electrical conduction through the dielectric spacer stack under the overlap region of the two electrodes. Current *I*_*E*_*(C*_*g*_*)* represents electrical conduction through the multiple molecular bridges formed along the edges of the top electrode. This current is a function of the gas concentration *C*_*g*_. In the absence of an analyte target gas (*C*_*g*_ = 0), current mainly flows through the spacer stack in the overlap region, with an overlap region of ~9 μm^2^; thus, *I*_*T*_ ≈ *I*_*S*_. On the other hand, when the target gas is captured between the nanogaps along the edges, current (*I*_*E*_*(Cg* > *0))* will additionally flow through the newly formed molecular junction in the edge undercut area of ~0.06 μm^2^. The device gap and linkers should be designed such that *I*_*E*_*(Cg* > *0)* ≫ *I*_*S*_ and *I*_*T*_ ≈ *I*_*E*_*(C*_*g*_*)* after the target gas is captured.

The total current flowing across the junction can be written as

1 $$I_T = I_S\left( V \right) + I_E\left( {V,C_g} \right)$$

where *I*_*T*_ is the total current flowing across the nanogap sensor and *V* is the bias voltage across the device. The substrate and edge current components have different functional forms. The substrate component, which corresponds to conduction through thin dielectric stacks, is readily modeled using the generalized Simmons’ formula for tunneling current^35^. The current (*I*_*S*_*)* across the overlap support region can be expressed as

2 $$I_S\left( V \right) = G_{SO} \cdot \left[ {\frac{{{\Phi} _s}}{q} \cdot e^{\left( { - 2 \cdot d_s \cdot \sqrt {\frac{{2m}}{\hbar^{2} } \cdot {\Phi} _s} } \right)} + \left( {\frac{{{\Phi} _s}}{q} + V} \right) \cdot e^{\left( { - 2 \cdot d_s \cdot \sqrt {\frac{{2m}}{\hbar^{2}} \cdot \left( {{\Phi} _s + qV} \right)} } \right)}} \right]$$

where Φ_*s*_ is the barrier energy of the spacer dielectric, *d*_*s*_ is the spacer layer thickness, *m* is the effective mass of tunneling electrons, *ħ* is the reduced Planck’s constant, and *q* is the charge of an electron. The parameter *G*_*SO*_ = *A*_*s*_*·g*_*SO*_ is a conductance fitting parameter proportional to the area, *A*_*s*_ = *W*^*2*^, of the spacer dielectric, and *g*_*SO*_ is a junction conductance factor.

The derivation of a mathematical expression for *I*_*E*_ is considerably more difficult^41,42^, and in general, it involves the calculation of nonequilibrium Green’s functions that specify the ballistic electron transport across the various molecular levels of the trapped gas molecule. Such calculations can lead to tunneling resonances and negative resistance regimes. The model that we have adopted below is based on our experimental observations, which did not display any resonant tunneling characteristics.

In the absence of a target gas, the *I*–*V* characteristics resemble those provided by a Simmons-like model. However, when investigating current conduction across molecular chains, Ghosh^43^ found that Simmons’ expression for the tunneling current was unable to describe the *I*–*V* characteristics accurately. The reason was that certain assumptions made during the derivation of Simmons’ expression did not hold for a molecular chain. N. Zimbovskaya^40^ found that the conduction through molecular junctions can be modeled by combining a Wentzel–Kramer–Brillouin (WKB) approximation of the transmission coefficient and the Landauer formula, which leads to a mathematical expression of the tunneling current similar to Simmons’ formula. The tunneling current depends exponentially on a function of the average energy barrier, as seen by the tunneling electrons. When the analyte gas is captured by our sensor, the dominant current is the gas-dependent edge current through the captured molecules, and using the Zimbovskaya formula, it is given by

3 $$I_{E}\left( {V,C_{G}} \right) = \left[ {\frac{{\beta \cdot C_{G}}}{{1 + \beta \cdot C_{G}}}} \right]\cdot {\Gamma} _{EO}\cdot \left[ \sqrt {\left( {\frac{{{\Phi} _E}}{q} + \frac{V}{2}} \right)} \cdot e^{ - 2 \cdot d_{E} \cdot \sqrt {\frac{{2 \ast m_E}}{{\hbar}^{2} }\cdot \left( {{\Phi} _{E} + \frac{{qV}}{2}} \right)} } - \sqrt {\left( {\frac{{{\Phi} _E}}{q} - \frac{V}{2}} \right)} \cdot e ^{ - 2\cdot d_{E}\cdot \sqrt {\frac{{2 \ast m_E}}{{\hbar}^{2}}\cdot \left( {{\Phi} _{E} - \frac{{qV}}{2}} \right)} } \right]$$

where *β* is a fitting parameter, *C*_*g*_ is the concentration of the target analyte gas in the test chamber, Φ_*E*_ is the average barrier potential of the hybrid molecular chain, and the distance *d*_*E*_ = 2 × (the length of the linker molecule) plus the length of the analyte molecule. Two parameters are expected to change in this equation when a gas molecule is captured. The first and most important gas concentration-dependent parameter is the average energy barrier Φ_*E*_, as shown in the zero-bias energy diagram of Fig. 5c. In Fig. 5c, Ψ_*SAM*_ and Ψ_*AU*_ are the work functions of the SAM layer and the gold electrode, respectively. *χ*_*target*_ is the electron affinity of the target gas.

Note that the average energy barrier depends on what is inside the edge gap. The energy barrier of the molecular bridge is obtained by fitting and comparing curves at different gas concentrations with the Zimbovskaya formula to obtain an estimate of the average energy barrier that electrons encounter. If no molecule is captured, then the average barrier is at the maximum given by the work function of the linker. If a molecule is captured, then the average barrier established on that junction depends on the location of the highest occupied molecular orbital (HOMO) and lowest unoccupied molecular orbital (LUMO) energy levels of the captured molecule. Curve fitting and parameter extraction reveal that in general, capture results in a lower average energy barrier Φ_*E*_ and hence a large enhancement of the device current.

The second concentration-dependent factor is the leading term

4 $$\left[ {\frac{{\beta \ast C_G}}{{1 + \beta \ast C_G}}} \right]{\Gamma} _{EO}$$

which is representative of the number of molecular junctions formed and is indicative of the saturation-type Langmuir adsorption characteristic^31^. The parameter *β* is a fitting parameter representing the adsorption characteristic, has a unit of 1/gas concentration and determines the low-concentration limit behavior of the edge current. This equation implies that the higher the surface concentration (C_G_ of the target gas) is, the larger the number of molecular junctions formed $(\beta \ast C_G)/(1 + \beta \ast C_G)$, ultimately leading to a higher edge current *I*_*E*_*(V,C*_*G*_*)*. The edge current saturates when all possible absorption sites are full. The parameter Γ_*E0*_ = *p*·*g*_*EO*_ is a fitting parameter proportional to the edge perimeter *p* = *4·(W* + *2*Δ*)*. This parameter specifies the magnitude of the nonlinear edge conductance in units of AV^−1/2^. Note that Eq. 3 also displays an exponential dependence of the molecular junction resistance on the total molecular length, in agreement with observations made by others^37,39,44^.

## Results and discussion

### Experimental *I*–*V* characteristics before and after gas exposure

**Fig. 6:**
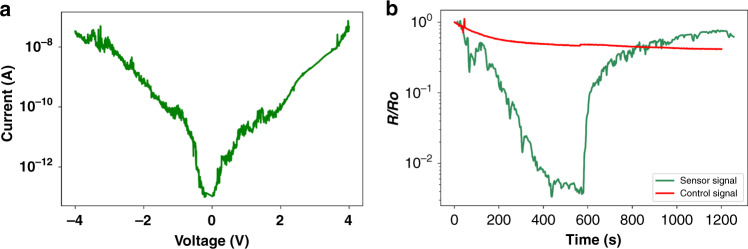
Electrical characterization of the fabricated device and response of sensor vs. control device. **a***I*–*V* curve of the fabricated device without gas exposure showing complete electrical isolation between upper and lower gold electrodes. **b** Sensor analysis over one complete cycle of exposure to 40 ppm cadaverine gas and its removal. The sensor signal is compared to the response of a ‘control’ chip, which is our nanogap device without proper functionalization exposed to 40 ppm cadaverine for the entirety of the experiment. *G/G*_*0*_ is the conductance of the sensor normalized to its lowest value, *G*_*0*_ (prior to gas exposure)

The fabricated sensor, without being exposed to a target gas, showed a minimal leakage current across the tunneling junction (on the order of ~50 pA with a measurement resolution of 0.1 pA) within a bias range of −4 V and 4 V, which indicated an average DC resistance of 74 GΩ, as shown in Fig. 6a. This result confirmed the ‘OFF’ state of the device before gas exposure, consuming an ultralow-power of pW in the standby condition. When the fabricated sensor was exposed to a target gas (cadaverine) at a concentration of 40 ppm, it exhibited a sudden reduction in the junction resistance or a sudden increase in the junction conductance of three orders of magnitude, clearly demonstrating switching to the ‘ON’ state, as shown in Fig. 6b. These results confirmed the initial physical disconnection and subsequent target capture-induced connection of an electrical path by molecular bridging, enabling high ON/OFF signal ratios as desired. The plot also shows the control (reference) chip signal (a device that was not coated with the linker molecule and exposed to similar ppm amounts of the target cadaverine gas). The feeble response of the control chip shows that in the absence of proper functionalization, even after exposure to cadaverine gas, there is a negligible sensor response, as thiol molecules are not present to capture the analyte molecules and the molecular switch remains in the ‘OFF’ state. Essentially, the lack of response from the control chip proves the validity of the linker molecules and their essential role in capturing the target gas molecules.

### Experimental and fitted model *I*–*V* characteristics at different gas concentrations

**Fig. 7:**
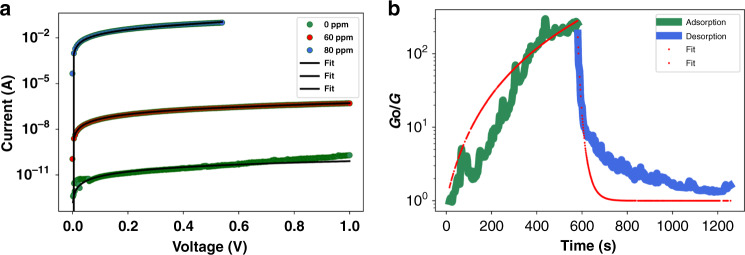
Curve fitting of sensor response to the modified Zimbovskaya model and Polanyi–Wigner equation. **a***I*–*V* measurements of the nanogap sensor device after successful capture of cadaverine molecules at various concentrations of the analyte curve-fitted to the established tunneling current models. The maximum root-mean-square error of the curve-fitting plots shown in the figure was found to be ~45%, 1%, and 0.6% of the average experimental data for the *I*–*V* characteristics of the nanogap junction after exposure to 0 ppm, 60 ppm, and 80 ppm cadaverine, respectively. **b** Normalized conductance response of the sensor to one period of exposure of the nanogap device to cadaverine and its subsequent removal, curve-fitted to adsorption-desorption models

Figure 7a shows plots of experimentally obtained *I*–*V* data curve-fitted to the tunneling current model, as described in Eq. 3, at various analyte concentrations and their tight agreement. The electrical measurements were taken after the sensor signal had reached a steady state. As evident from the plot, exposure to ~80 ppm cadaverine led to a reduction in the junction resistance by ~9 orders of magnitude. The capture of cadaverine molecules is facilitated by the electrostatic attraction between the end benzoic acid group of the linker molecule and the highly polar NH_2_ end group of the cadaverine molecule. Additionally, the nano-air-gap dimension is designed to be ~6 nm. The linker molecule is ~2.5 nm in length. Therefore, after proper functionalization, the nano-air-gap dimension reduces to ~1.0 nm, making it ideal for capture of cadaverine since its length is ~1.0 nm. We believe that the electrostatic attraction combined with careful fabrication of the nanogap is the reason cadaverine is captured and thereby detected strongly. Successful capture is accompanied by the formation of hydrogen bonds between the linker and cadaverine molecules. According to the phenomenological model provided by Zimbovskaya^40^, capture of cadaverine molecules leads to a decrease in the average potential barrier of the tunneling junction, which allows for an augmented electron flow across it. The tunneling current is an exponential function of the potential barrier encountered by electrons^45^. Therefore, even a slight decrease in the potential barrier leads to a significant increase in the tunneling current, which is observed as a strong signal upon capturing cadaverine.

Theoretical parameter extraction demonstrated that the exposure of the fabricated device to ~80 ppm cadaverine gas reduced the average potential barrier from ~5 eV to ~0.9 eV, resulting in exponential tunneling current variation with the square root of the barrier potential change, and that the fitting parameters *β* and Г_E0_ in Eq. 3 were 21.685/ppm and 0.3688 A/V^−1/2^, respectively. It must be noted that the model-extracted values of the reduced average potential barrier were not a quantitative measurement of the HOMO-LUMO levels of individual target/linker molecules but a cumulative indication of the average potential barrier faced by tunneling electrons. In fact, Zimbovskaya^40^ mentioned that the inherent disadvantage of this mathematical model is that although one can accurately describe *I-V* characteristics using Eq. 3, detailed information, including the electrostatic potential profile of the transport channel, cannot be obtained. This is because of the WKB approximation for electron transmission functions used to derive the model in the first place.

### Transient response characteristics: adsorption-desorption dynamics

Measurement results indicated that the dynamics of a junction resistance drop were governed by a quasi-irreversible process of cadaverine molecules forming hydrogen bonds with the linker end group and producing a hybrid molecular bridge. Such a typical chemisorption process had been previously described mathematically by Elovich’s equations, and the desorption process can be described by the Polanyi–Wigner equation^46^, which describes the adsorbed gas molecule being released back into a gaseous form, ideally consisting of complete breaking of hydrogen bonds between the target gas molecules and the linker end group. Figure 7b shows the normalized conductance versus time plot of one complete cycle of exposure of the device to the analyte and its subsequent removal, curve-fitted with the Elovich equation for the adsorption cycle and Polanyi–Wigner equation for the desorption cycle. The plot suggests that the mathematical model was in decent agreement with the experimental data.

### Selectivity of the gas response

**Fig. 8:**
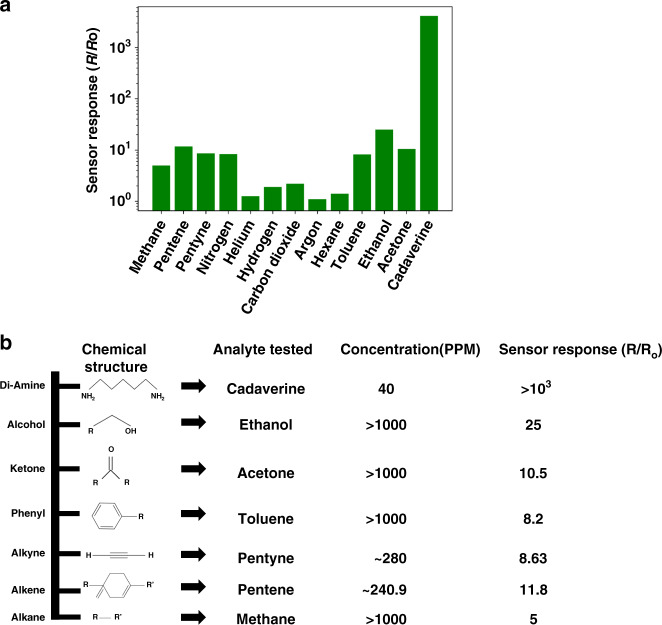
Selectivity studies of the nanogap sensor. The sensor was exposed to multiple gases and analytes with different molecular end groups to determine its cross-selectivity. **a** A comparitive bar-graph showing the response of sensor to different analytes. **b** Device response to commonly found gases and analytes,their concentration and molecular molecular end groups.

To investigate the cross-sensitivity of the sensor, we exposed the device to analytes with different molecular end groups, commonly found VOCs and gases such as helium, hydrogen, and CO_2_. We define the sensor response as the normalized junction resistance drop in the steady-state after exposure to the analyte. Figure 8a shows the sensor response when exposed to these analytes compared to that when exposed to the intended target gas, cadaverine. Figure 8b shows the quantitative response of the nanogap sensor when exposed to analytes with different molecular end groups. Measurements revealed an R_OFF_/R_ON_ ratio of more than 3 orders of magnitude when exposed to only 40 ppm cadaverine, whereas a maximum R_OFF_/R_ON_ ratio of ~1 order of magnitude was observed when exposed to much greater concentrations of the other analytes. The concentration of the VOCs and other gases was maintained at greater than 1000 ppm. The response to moisture was ~2 orders of magnitude; however, the relative humidity was maintained at ~80% (~20,000 ppm). To measure the device response in the presence of other gases, we flooded the test chamber with the specific test gas and then monitored the resistance drop of the sensor. After observing the steady-state response to a certain target gas, the sensor was removed from the test chamber, and the analyte molecules were allowed to naturally escape. This unassisted recovery was monitored electrically by continuously probing the junction resistance of the sensor, and complete recovery was assumed to have been achieved when the junction resistance was measured to be at its original value (as measured before exposure to the gas). Following this, the sensor was subsequently exposed to the next target gas. These results suggest highly selective sensor action against the most commonly found VOCs. The limited sensitivity to other gases may be due to smaller molecules or even moisture reducing the air gap between the electrodes due to chemisorption.

## Conclusions

We present a new class of chemiresistors based on the capture of gas molecules within a nanoscale gap. We fabricated gas-sensing devices with gold electrodes separated by an ~6 nm nanogap functionalized with a fully conjugated terphenyl linker molecule (4-((4-((4-mercaptophenyl)ethynyl)phenyl)ethynyl)benzoic acid) for electrostatic capture of cadaverine gas. We demonstrated ultralow-power resistance switching in batch-fabricated nanogap junctions upon detection of the target analyte–cadaverine. The stand-by power consumption was measured to be less than 15.0 pW, and the R_OFF_/R_ON_ ratio was more than eight orders of magnitude when exposed to ~80 ppm cadaverine. A phenomenological electrical model of the device is also presented, in good agreement with experimental observations. The cross-sensitivity of the gas sensor was tested by exposing the device to some of the commonly found VOCs and other atmospheric gases. The experiments revealed highly selective sensor action against most of these analytes. These batch-fabricated sensors consume ultralow power and demonstrate high selectivity; therefore, they are potential candidates for sensor application in power-critical IoT applications and can fulfill other low-power sensor needs. Given the dynamic response of the sensor, we believe that it can be used for quantitative measurements proportional to the target gas concentration. This enhances the possible impact of this technology toward the development of selective sensors based on this detection principle.

## Supplementary information

SUPPLEMENTARY MATERIAL
