# Supplementary material for: Molecular bridge-mediated ultralow-power gas sensing
Source: Microsyst Nanoeng. 2021 Mar 29;7:27. doi: 10.1038/s41378-021-00252-3 (PMC8433217; doi:10.1038/s41378-021-00252-3)
Supplement: Supplementary file 1 — SUPPLEMENTARY MATERIAL [file 41378_2021_252_MOESM1_ESM.docx]

**SUPPLEMENTARY MATERIAL**

**Molecular Bridge-Mediated Ultralow-Power Gas Sensing**

Aishwaryadev Banerjee^1*^, Shakir-Ul Haque Khan^1^, Samuel Broadbent^2^, Ashrafuzzaman Bulbul^1^, Kyeong Heon Kim^3^, Seungbeom Noh^1^, R. Looper^2^, C. H Mastrangelo^1^ and H. Kim^1^

1 Department of Electrical and Computer Engineering, University of Utah, Salt Lake City, UT 84112, USA; aishwaryadev.Banerjee@utah.edu (A.B.); khanshakirul@gmail.com (S.H.K.); a.bulbul@utah.edu (A.Bu.); moses.noh@utah.edu (S.N.); hanseup@gmail.com (H.K.), carlos.mastrangelo@utah.edu (C.H.M.)

2 Department of Chemistry, University of Utah, Salt Lake City, UT 84112, USA; samuelbroadbentnj@gmail.com (S.B.); r.looper@utah.edu (R.L.)

3 Gyeongsang National University; khkim0124@gmail.com (K.H.K.)

* Corresponding Author: aishwaryadev.banerjee@utah.edu; Department of Electrical and Computer Engineering, University of Utah, Salt Lake City, USA

***Target Molecule and SAM Capture Linker Synthesis***

*Capture Linker Synthesis*: The linkers, consisting of three benzene rings (Figure S1-right), were synthesized to be able to capture amine group molecules (Figure 1-left). Figure S2 shows the chemical synthesis process starting from commercially available 4-iodobenzoate (1) that first underwent a Sonogashira reaction with trimethylsilyl acetylene to introduce the first alkyne (2). Next, the alkyne was freed by removing the protecting group (3) and subsequently went through another Sonogashira with 1-Bromo-4-Iodobenzene to introduce the second phenyl ring on the free alkyne (4). Identical steps were repeated to add the second alkyne to the second phenyl ring and to remove the protecting group from the alkyne to yield the free alkyne terminal (5, 6). As the coupling partner to the free alkyne terminal, commercially available 4-iodobenzenesulfonyl chloride (7) was reduced to the sulfonic acid and converted to the corresponding thioacetate (8) in order to prevent a free thiol from poisoning the palladium in the final Sonogashira coupling. Then, these two compounds were coupled via a final Sonogashira reaction to provide the fully conjugated ter phenyl linker (9). The thioacetate part of the coupled compounds were cleaved through saponification using lithium hydroxide to yield the final linker molecule. During the process the methyl ester was converted to the corresponding carboxylic acid that was designed to capture the target amine group molecules (10). The thiol end-group of the molecule covalently bound to the exposed gold surface of the electrodes, forming an Au-S bond as a firm linker coating.

|  |
| --- |
|  |

|  |
| --- |
|  |
| **Figure. 2** **a)** Synthesis of left and right fragments of linker molecule **b)** coupling of the left and right fragments to form the thiol-linker molecule. |
